# Assessing Food Preferences and Neophobias among Spanish Adolescents from Castilla–La Mancha

**DOI:** 10.3390/foods12203717

**Published:** 2023-10-10

**Authors:** Carmen del Campo, Cristina Bouzas, Margalida Monserrat-Mesquida, Josep A. Tur

**Affiliations:** 1 Research Group in Community Nutrition and Oxidative Stress, University of the Balearic Islands-IUNICS, 07122 Palma de Mallorca, Spain; 2 CIBER Fisiopatología de la Obesidad y Nutrición (CIBEROBN), Instituto de Salud Carlos III (ISCIII), 28029 Madrid, Spain; 3 Health Research Institute of Balearic Islands (IdISBa), 07120 Palma de Mallorca, Spain

**Keywords:** food preferences, taste, food neophobias, food habits, adolescents, Spain

## Abstract

Food neophobia is a reaction of dislike or fear of food, which may be due to a wide variety of factors (taste, texture, exposure at an early age, genetics, or diversity in feeding practices and food consumption). The aim of this study was to assess the preferences for tastes and foods and food neophobias among Spanish adolescents and to compare the differences between boys and girls. This was a cross-sectional observational study on 11–18-year-old healthy adolescents (n = 600; 50% female) recruited in the Castilla–La Mancha region (central Spain). Information on taste preferences, food neophobias, anthropometric measurements, and sociodemographic data was recorded. The highest taste preference was found for sweet, salty, and umami. Most adolescents usually did not try new foods outside the home, nor did they like to try foods from other countries. More than half of them also acknowledged being selective eaters or were very particular about the foods they ate. There were no significant associations between taste preference and neophobias with obesity, waist-to-height ratio (WtHR), sleep, and smoking. Adolescents showed a high unwillingness to change food habits, and most of the food neophobias found in the current study were related to new, novel, or previously unknown foods. Spanish adolescents from central Spain (Castilla–La Mancha region) showed a preference for sweet, salty, and umami tastes of foods, as well as food neophobia towards foods that they do not regularly consume, mainly those with a bitter taste. Gender and body weight showed little influence, and age had a moderate influence on food neophobias. Familiarity with foods, as well as educational activities, are suggested as useful to decrease food neophobias among adolescents.

## 1. Introduction

The World Health Organization defines food neophobia as a reaction of dislike or fear to a food or a food group [1], which is characterized by a reluctance to eat new or unfamiliar foods and has been linked to a reduction in the variety and quality of the diet [2]. Food neophobia, from an evolutionary point of view, may be an innate behavior to avoid or to minimize risks of eating foods that are harmful to health [3,4,5]. However, food neophobia can cause eating monotony, which can result in nutritional deficiencies. The low variety of nutrients caused by food neophobia may restrict the intake of nutrients needed to maintain the body’s homeostasis. When this restriction is severe and/or prolonged for a long time, there will be dysfunction of several systems in the organism, as well as alteration of the normal development of children and adolescents [3,6].

The mechanism of the onset of food neophobia is not fully understood. It may be due to a combination of biological, psychological, and environmental factors. These include individual, cultural and religious predispositions, preference or rejection of a taste (i.e., salt, sweet, or bitter) as well as overall flavor perception, where taste and odor are combined, familiarity with a taste or a smell, texture (crunchy when raw, soft after excessive cooking or fibrous), the moment and method of introducing new products, exposure to foods at an early age, ignorance or negative experience when trying these foods, either due to an inadequate diet or cooking or due to a family environment not conducive to a particular food’s consumption, parents’ attitude towards foods, and physiological conditions (health status) or genetic factors which are still being studied [7,8,9,10,11,12].

Food neophobia may be due to a previous negative experience with that food, such as an allergic reaction or intolerance, or an innate aversion, even without prior contact [13,14]. Food neophobias can be mild or severe and temporary or long-lasting [3]. In some cases, food aversions can be treated with cognitive behavioral therapy, but due to the influence of environmental factors and the emotional component, they usually appear in childhood [2] and usually decrease in adolescence, but they can be present in adults [15]. The main reason for neophobia is related to the fear of trying new or unknown foods that a person has not eaten before or that are not typical of their culture [3].

Food neophobia can affect the dietary variety and hedonic acceptance due to the rejection of healthy foods [16]. The most usual food neophobias include aversion to meats, greens, and vegetables. Some people may have phobias about certain meats, such as beef or game, due to their taste or environmental concerns. This is one of the possible reasons for the current dietary trend towards following vegetarian or vegan diets [17,18,19]. Other people may have phobias about green or plant-based foods due to their taste or texture, but the main cause is a psychological phobia without prior contact with the food [2,3,20,21]. Aversion to foods can have consequences on the nutritional status of the individual and may be due to a wide variety of factors. The confluence of poor food choices and the exclusion of healthy foods, either due to lack of access to them or due to innate aversion and predisposing environmental factors, may contribute to several dysfunctions and loss of health [3].

Diversity in feeding practices and food consumption could be considered another factor contributing to food neophobias. Until now, a pan-European study in children and adolescents was carried out, finding different levels of food neophobia between countries [22]. These differences in food neophobias among children may be due to different food cultures and may be ascribed to differences in feeding practices and different food availability in the assessed countries [22]. Women are super-tasters. This means that women are more likely to perceive more accurately different flavors than men. This could be partially explained because it was pointed out that women have a higher density of taste receptors, as they have more fungiform papillae, which are responsible for the accuracy and intensity of taste perception [23]. This should be taken into consideration when studying food neophobia.

Spain is considered a Mediterranean country from a dietary point of view [24]. However, there are cultural differences regarding the characteristics and common dishes and recipes in the different Spanish regions [25]. Food neophobias were assessed among Spanish adolescents living in the Basque Country [10,11] and Murcia region, located in Southeastern Spain [26], but not in other Spanish regions. The aim of this study was to assess the preferences for tastes and foods and food neophobias among Spanish adolescents and to compare the differences between boys and girls.

## 2. Methods

### 2.1. Design and Sample Recruitment

A cross-sectional observational study was conducted on 11–18-year-old healthy adolescents (population 226,734 adolescents; sample n = 600; 50% female) recruited from three secondary school centers in the Castilla–La Mancha region, Spain, in February 2022. The sample size was calculated with a statistical power of 95%, accepting an alpha risk of 0.05, a beta risk of 0.2 in a two-sided test, a common deviation assumed as 1.4, and anticipating a drop-out rate of 10%, 210 subjects per gender were necessary to be statistically significant. To be conservative, 300 participants per gender were used, with a final sample of n = 600. Schools were randomly selected among the available schools in the region. In each school, a class or two were randomly selected to answer the questionnaires (see Supplementary Material). Surveys were completed on paper and then implemented into a database. Information on food preferences and neophobias, anthropometric measurements, and sociodemographic data were recorded.

This study was approved by the Medicinal Research Ethics Committee of the Ciudad Real General University Hospital, Spain (ref. C-498), and the written consent of parents and children was obtained. The guidance department of the secondary school centers was present at the time the measurements and the surveys were carried out.

### 2.2. Anthropometrics and Sociodemographic Data

Anthropometric measurements by gender were made according to standard procedures, namely that the participants were dressed in light clothing and barefoot and were taken into a room separate from the classroom to preserve the adolescents’ privacy. Height was measured to the nearest millimeter, with the participants’ heads maintained in the Frankfurt horizontal plane, using a mobile stadiometer (Seca 213, SECA Deutschland, Hamburg, Germany). Body weight (kg) was measured with a bioimpedance device (Inbody 120, Microcaya, Bilbao, Spain). Body mass index (BMI) was calculated as body weight (kg) divided by the square of body height (m^2^). BMI standard deviation scores were calculated using age and gender-specific WHO reference values [27], and the 85th and the 97th percentile were used to define overweight and obesity, respectively. Waist circumference was measured twice using an anthropometric tape (Seca 201, SECA Deutschland, Hamburg, Germany) in a standing position midway between the last rib and the iliac crest. The mean of the two measurements was recorded to the nearest millimeter. The waist-to-height ratio (WtHR) was calculated from the anthropometric data collected on waist circumference and height. A cut-off >0.5 was used to define abdominal obesity [28]. Sociodemographic data (age, sleeping time, smoking, food allergies, and intolerances, as well as social, cultural, and religious habits) were also registered.

### 2.3. Preference for Food Taste Assessment

Preference for the five food tastes (sweet, salty, bitter, sour, and umami) was assessed by gender and age by applying the Hedonic Food Scale [29], which measures the subjective preference of an individual towards foods. Both were used according to the following scale (range 1–9): dislike extremely (value 1), dislike very much (value 2), dislike moderately (value 3), dislike slightly (value 4), neither like nor dislike (value 5), like slightly (value 6), like moderately (value 7), like very much (value 8), and like extremely (value 9). Association between preference for tastes and obesity, waist-to-height ratio (WtHR), sleep, and smoking were assessed by gender and age.

### 2.4. Food Neophobia Assessment

The Pliner and Hobden Food Neophobia Scale (FNS) [30] was used to assess the fear of trying foods by gender, which is based on ten questions about the frequency of trying new foods, which were evaluated using a 5-point Likert scale ranging from 1 = “I strongly disagree” to 5 = “I strongly agree”. Items 1, 4, 6, 9, and 10 were reversed. The score theoretically ranged from 10 to 50, and the highest score reflected the highest neophobic level. The unwillingness to change regarding trying new foods in the future was calculated from the summatory of the FNS items. This scale may range from 0 to 5, as it is the result of processing the questionnaire.

Neophobia towards food groups (grains and tubers, vegetables, fruits, nuts, pulses, diaries, meats and vegetable substitutes, and eggs) was assessed by gender by applying two possible responses: “I like it” vs. “I do not like it”.

Particular controversial foods were assessed by gender with multiple-choice items based on four possibilities: “I tried it, and I like it”, “I tried it, and I do not like it”, “I did not try it“, and “I did not try it, and I don’t like it”. That last answer was useful to assess neophobia for psychological reasons.

### 2.5. Statistics

The SPSS statistical software package version 27.0 (SPPS Inc., Chicago, IL, USA) was used for analysis. Data are shown as mean and standard deviation (SD) except for prevalence data that were expressed as percentages. A chi-squared test was calculated for categorical variables. For continuous variables, a Student *t*-test (differences between genders) and a one-way ANOVA (differences between ages) were used. Logistic regression was fitted for associating each one of the items of preference for flavors and food neophobia after adjustment by sociodemographic data.

## 3. Results

The characteristics of the respondents are shown in Table 1. Boys showed higher body weight, height, and waist circumference, as well as a prevalence of being overweight, obesity BMI, abdominal obesity, and sleeping daily (more than 8 h/day) than girls. There are more girls smoking than boys. The population with food allergies was low. The most common allergies were those to fruits, nuts, fish, and eggs and were more common among boys than girls. Lactose intolerance was highest among adolescents, but scarce intolerance to gluten and fructose was observed, both higher among boys than among girls.

Table 2 shows the preference of adolescents for food taste by gender. The highest preference was found for sweet, salty, and umami. Boys showed a higher preference for sweet and bitter than girls (Table 3). There were no significant associations between preference for taste and obesity, WtHR, sleep, and smoking.

Table 4 shows the preference of adolescents for food taste by age. The highest preference was found for sweet, salty, and umami. Table 5 shows that the highest preferences for sweet and lowest for bitter were found in 16–18-year-old adolescents; 16–18-year-old girls showed the highest preference for umami.

Food neophobias among adolescents by gender are shown in Table 6. Most adolescents usually did not like to try new foods, nor did they try new foods outside the home, nor liked to try foods from other countries. More than half of them also acknowledged being selective eaters or were very particular about the foods they ate. More girls than boys did not like foods from other cultures or ethnicities and declared themselves very particular about the foods they ate and did not eat almost anything. Table 7 shows the association between age and food neophobias. The older adolescents are, the more reluctant they are to try new foods. The Food Neophobia Scale (FNS) changed according to the age of the adolescents: In boys, it was 2.60 ± 0.76 (11 years old), 2.56 ± 0.75 (12 years old), 2.45 ± 0.69 (13 years old), 2.41 ± 0.70 (14 years old), 2.25 ± 0.70 (15 years old), 2.52 ± 0.65 (16 years old), 2.59 ± 0.65 (17 years old), and 2.89 ± 0.46 (18 years old), with a *p*-value = 0.031 (by ANOVA) for trends. In girls it was 3.33 ± 0.78 (11 years old), 2.64 ± 0.87 (12 years old), 2.36 ± 0.73 (13 years old), 2.48 ± 0.83 (14 years old), 2.32 ± 0.79 (15 years old), 2.58 ± 0.62 (16 years old), 2.64 ± 0.64 (17 years old), and 2.78 ± 0.45 (18 years old), with a *p*-value = 0.159 (by ANOVA) for trends. Food neophobias were not related to the prevalence of overweight, obesity, abdominal obesity, sleeping daily (more than 8 h/day), and smoking.

Table 8 shows the association between preferences for food tastes and the ten items of the Food Neophobia Scale. The main findings of this table are that the preference for sour and umami flavors is associated with lower food neophobia. Items pro-neophobia were inversely associated with sour and umami, whilst no-neophobia items were directly associated with those tastes. A preference for sweet was only relevant in the acceptance of foods from other countries or cultures. Liking bitter was associated with a higher likelihood of eating almost any kind of food. Moreover, participants who declared preferences for bitter, sour, or umami flavors were those who were more likely to be predisposed to change or try foods.

Table 9 shows the association between gender and neophobia towards food groups. Girls showed higher neophobia towards vegetables and lower to meats and vegetable substitutes than boys. Table 10 shows the association between age and food neophobia towards food groups. The older the adolescents are, the higher the acceptance of grains and tubers and fruits, and the lower the vegetables and pulses.

**Figure 1:**
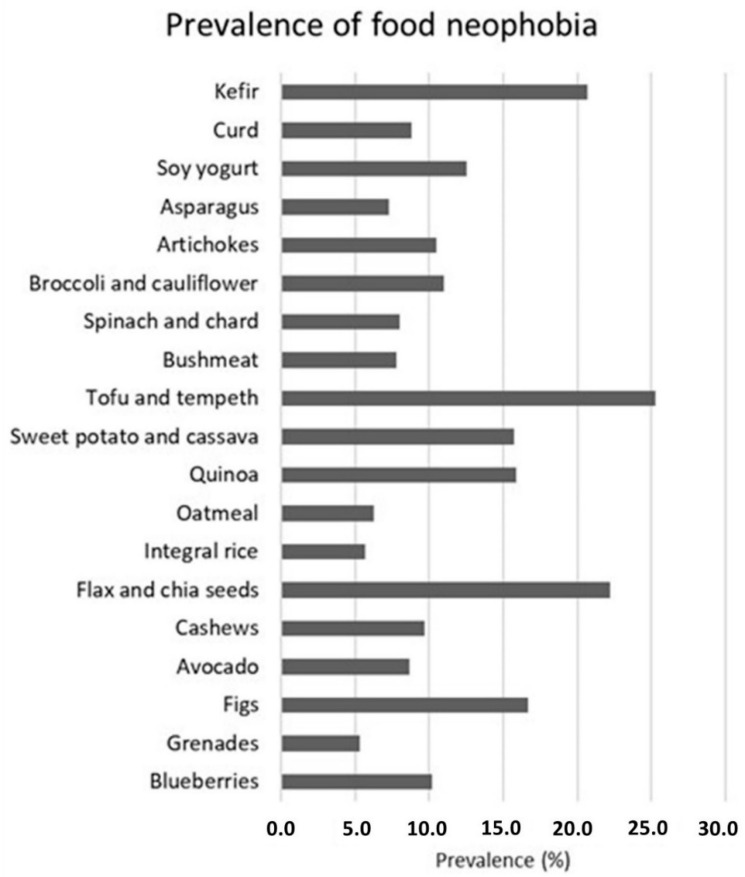
Prevalence of food neophobia.

Adolescents showed no neophobia for most fruits (strawberries, bananas, peaches, pineapples, and pomegranates). Neophobia was observed for figs, blueberries, and avocados. Boys showed neophobia for bananas and peaches more than girls. No neophobia was observed for most of the nuts (peanuts, almonds, walnuts, and pistachios). Flax and chia seeds were mostly refused by adolescents, showing neophobia. Whole grains (wholemeal bread, integral rice, integral pasta, and oatmeal) showed low neophobia among adolescents; however, quinoa was intensively refused by adolescents. Potatoes were mostly accepted by adolescents, but sweet potatoes and cassava were mostly refused. Eggs were equally accepted by boys and girls. Pulses (chickpeas, lentils, and peas) showed no neophobia, but beans were highly refused, more so among girls. Among meats, bushmeat and butt showed neophobia; neophobia to tofu and tempeh was found among girls, and neophobia to bushmeat was higher among boys. Vegetables (green leafy, spinach and chard, tomato and cucumber, aubergines and zucchini, broccoli and cauliflower, artichokes, and asparagus) were accepted by adolescents; however, neophobia was registered for broccoli and cauliflower, spinach and chard, and artichokes. Dairy products (milk or vegetable smoothies, sheep’s cheese, yogurt, and goat’s cheese) were highly accepted by adolescents; however, the newest dairy products (soy yogurt, curd, and kefir) were highly refused, with adolescents showing neophobia for them (Figure 1).

## 4. Discussion

The current study showed that Spanish adolescents from Castilla–La Mancha (central Spain) had the highest preference for sweet, salty, and umami but the lowest for bitter and sour. The adolescents did not display neophobia to most fruits, nuts, whole foods, potatoes, eggs, and most legumes, vegetables, and dairy products. Neophobias were observed in figs and avocados, flax seeds and chia, quinoa, sweet potatoes and cassava, beans, wild meat, tofu and tempeh, broccoli and cauliflower, spinach and chard, and artichokes, as well as newer dairy products (soy yogurt, curd, and kefir).

These current results agree with previous studies that showed that neophobic adolescents consumed fewer fruits and vegetables, mainly those with a bitter taste [2,15,17,24,26,31,32,33,34,35], and that food neophobia was associated negatively with fruits, vegetables, and whole foods in children [22]. The current results are also consistent with previous data showing that the predilection for sweet and salty tastes was innate, while the aversion to bitter and sour tastes is a factor associated with food neophobia [3]. Children and adolescents generally showed a low level of acceptance of food products with predominantly bitter or sour tastes, which may contribute to the formation of neophobias towards various foods, mainly those with bitter tastes [9,12,36]. Since the acceptability of food products is a subjective measure based on hedonism or pleasure influenced by the organoleptic properties of foods [37], flavor perception is highly determinant [38].

The current preference for sour and umami tastes was inversely associated with proneophobic elements and directly with non-neophobic elements, and the preference for sweet taste was relevant in the acceptance of foods from other countries or cultures. Children are born with the ability to taste, smell, and discriminate foods and to learn to appreciate new foods and organoleptic properties. However, reactions to tastes can hinder these processes, and rejection of specific foods can be associated with various tastes [39]. A liking for vegetables and beverages characterized by high levels of warning stimuli (i.e., bitterness and sourness) was lower among high neophobics, especially if subjects perceived the tastes of the novel foods as unpleasant and potentially dangerous [40]. The development of food preferences in relation to taste exposure occurs in early childhood when chemosensory senses become functional, and exposure to new foods is likely to accept new tastes and food preferences [41,42].

It was pointed out that vegetable consumption among adolescents depends on each vegetable and cannot be generalized [11], but there is full agreement that personal, behavioral, and environmental factors are involved in vegetable consumption [43]. Within these factors, the taste characteristics of vegetables and consumption habits in the family environment play an important role in accepting or rejecting vegetables. Vegetables characterized by a bitter and astringent taste were less appreciated, while sweet and mild vegetables were more accepted [11,31,35,44,45,46]. The adolescents described tomatoes, green beans, peas, potatoes, and zucchini as sweet and mild vegetables, and they accepted them. In contrast, broccoli, cauliflower, spinach, chard, and artichokes were described as bitter and astringent, and they were rejected. However, when the rejected vegetables are commonly consumed in the region where the adolescents lived [47], such as the consumption of asparagus in the present study among adolescents from Castilla–La Mancha, these vegetables were accepted due to the high familiarity of the adolescents with this food [17,40], which is in accordance with the well-assumed knowledge that food neophobia is negatively associated with the acceptance not only of new or unfamiliar foods but also of familiar foods [48]. Therefore, the treatment of food neophobia should include familiarization with new foods and their appearance, taste, texture, and systematic introduction [8,41].

Familiarity is a prominent motivator in food choices; however, food neophobia is not an immutable personality trait [48]. In this sense, participating adolescents showed a high reluctance to change their eating habits, and most of the food neophobias found in the present study were related to new, novel, or previously unknown foods. It was previously reported that food neophobia is related to the willingness or unwillingness to try new foods [49,50], creating positive or negative experiences with new flavors [51]. The current study showed that preferences for sour, bitter, or umami are related to a greater willingness to try new foods and be exposed to foods they had neophobia for. Furthermore, a positive correlation was found between food neophobia and negative reactions to new stimuli, and even not to food stimuli [3,52,53]. Adolescents who had a higher level of neophobia feared novelty, thus avoiding foods with unfamiliar tastes, colors, or textures [3].

It was also suggested that place of residence, social setting, cultural influences, food context, and the availability of different foods are determinants of food choice, as well as food neophobia or the refusal to eat unfamiliar foods [3,38,54,55]. Adolescents in this study live in Castilla–La Mancha (central Spain), a rural area, and are accustomed to a cuisine with a wide variety of traditional, hearty but simple dishes, which are prepared with basic ingredients such as bread, meat, vegetables, and sheep’s cheese [24]. This region is far from the most touristy Spanish regions (i.e., the Balearic Islands, Canary Islands, Catalonia, and southern Andalusia), where novel and ethnic foods are more common in markets and restaurants [56]. Therefore, the low availability of new foods in rural areas may explain the lack of willingness to try new foods among the adolescents currently studied [32,57].

Intervention studies suggested that educational programs based on food-related activities to increase familiarity and exposure to foods and create positive attitudes and experiences with new foods can decrease food neophobia [49]. In this sense, it was pointed out that one way to increase experiences with new tastes could be to introduce sensory education lessons as part of school programs, which were carried out in different countries with positive results by increasing variety in diets [58,59]. Therefore, introducing new tastes into the diet will increase future food preferences, avoiding future food neophobias. A clinical trial conducted in schools in northern Spain demonstrated that diet quality can be improved through an intervention focused on engaging children in cooking activities at home [60]. These results allow us to deduce that people with high food neophobia need additional support to improve the quality of their eating behavior and, therefore, the health quality of their diet [48]. Therefore, it is necessary to encourage nutritional education plans to increase vegetable consumption in children and adolescents to make vegetables tastier and easily accessible [43].

Fear of gaining weight could explain the refusal to eat unfamiliar foods among adolescents [61], but previous results showed that food neophobia was not related to either BMI or body fat percentage [10,11,62,63,64]. However, other authors reported a decrease in BMI with food neophobia [65,66,67,68,69,70]. Previous authors reported that slowness of eating [71,72,73] and responsiveness to satiety [71,72] was greater in girls, and restlessness about food was greater in boys, highlighting the greater concern about the regulation of weight and eating habits in women. Despite these previous results, food neophobia was not related to obesity in the adolescents currently evaluated. More research would be needed to clarify the relationship between fear of gaining weight and food neophobia, especially if this relationship were linked to gender.

Few differences were found in food neophobia between boys and girls in the current study: boys showed a higher preference for sweet and sour and neophobia for tofu and tempeh, as well as bananas and peaches, than girls, who showed neophobia for bushmeat and did not like wild meat, or foods from other cultures or ethnicities. Previous results on the differences between genders are controversial. Thus, food neophobia was reported to be more common among women than men [10,74,75]; other studies found neophobia among men [49,50], and other boys and girls did not differ in their food neophobia [76]. Furthermore, no significant differences were shown in relation to gender scales commonly used to quantify food neophobia [75,77,78,79]. Therefore, more research is needed to clarify the relationship between gender and food neophobia.

Current results showed that the occurrence of food neophobia was similar across age groups of adolescents, something higher in boys than in girls, showing just minor differences in taste preferences (sweet, bitter, and umami) between ages, which agrees with previous findings, as well as that food neophobias were slightly higher in boys [10,80,81]. The current results also showed that the older the adolescents were, the higher the acceptance of several foods (grains and tubers and fruits), and the lower of others (vegetables and pulses), despite it being pointed out that food neophobia decreases around the age of five [10], which resulted in the rejection to try new foods as they got older. It suggests that other determinants of food choice (place of residence, social setting, cultural influences, food context, and food availability) are more powerful than age among adolescents. No other significant associations between measured variables and sociodemographic factors and other variables were found.

The current results suggest that food neophobia may be an important risk factor for poor nutritional status among children and adolescents because it is a period of special vulnerability. Childhood and adolescence are periods of life with extensive neuroanatomical and functional reorganization of the brain, which occurs in parallel with substantial maturational changes in behavior and cognition [82]. Therefore, food neophobia could be the cause of low adherence to healthy dietary recommendations and then inhibit the adaptation of healthy and sustainable diets among children and adolescents. In this sense, food neophobia could affect adherence to the Mediterranean diet, a quality eating pattern [1], and the acceptance of healthy foods. Future research should attempt to understand the implications of food neophobia on healthy eating behavior. Several therapies were suggested to reduce food neophobias in children and adolescents. The role of the family is essential to decrease food neophobias, but it is more effective in children; in adolescents, it is recommended to reduce anxiety, introduce a therapy of desensitization, and introduce new foods progressively and associated with positive experiences. These therapies should be supervised by a physician or a psychologist [81].

After the current results were analyzed and compared with previous data, it can be concluded that body weight and gender have little influence on food neophobias in adolescents. Age has a moderate influence on food neophobias in adolescents and can be corrected by means of educational programs. Food tastes have the highest effect on the appearance of food neophobia, mainly if a bitter taste is associated with these foods [2,8,9,12,15,17,24,26,31,32,33,34,35,36,39,41]; however, other determinants, like familiarity with foods, place of residence, social setting, cultural influences, food context, and the availability of these foods [8,41,48], as well as parents’ educational roles [41,42] are essential to avoid or to reduce food neophobias.

## 5. Strengths and Limitations

The current study provides information on the taste preferences and food neophobias of Spanish adolescents residing in Castilla–La Mancha. The main strength of the study is its large sample size. Furthermore, the main limitation is that this study was observational and cross-sectional, and its design does not allow causal inferences to be established; therefore, only associations were made. Another limitation was the validity and reliability of the dietary intake data from the surveys, its potential for bias being well-known [83]. However, because the questionnaires did not address the consumption of specific foods, the risk of bias in whether to try a particular food is lower. An additional limitation is that the present sample is not necessarily representative of the entire Spanish child and adolescent population. Consequently, the results obtained now need to be corroborated with a larger sample.

## 6. Conclusions

Spanish adolescents from central Spain (Castilla–La Mancha region) showed a preference for sweet, salty, and umami tastes of foods, as well as food neophobia towards foods that they do not regularly consume, mainly those with a bitter taste. Gender and body weight showed little influence and age-moderate influence on food neophobias. Familiarity with foods, as well as educational activities, are suggested as useful methods to decrease food neophobias among adolescents.

## Figures and Tables

**Table 1:** Characteristics of the respondents.

	All (n = 600)	Boys (n = 300)	Girls (n = 300)	*p*-Value
Age (yr)	14.2 ± 1.6	14.3 ± 1.6	14.2 ± 1.6	0.539 *
Age groups (%)				0.407 ^#^
11–13 year-old	37.3	35.0	39.7	
14–15 year-old	35.3	37.7	33.0	
16–18 year-old	27.3	27.3	27.3	
Weight (kg)	59.0 ± 13.9	62.7 ± 14.6	55.3 ± 10.9	<0.001 *
Height (cm)	164.8 ± 9.2	169.1 ± 9.5	160.6 ± 6.4	<0.001 *
Body Mass Index (kg/m^2^)	21.6 ± 3.9	21.8 ± 4.1	21.4 ± 3.7	0.003 *
Overweight (%)	20.2	23.4	16.7	0.005 ^#^
Obesity (%)	7.0	9.4	4.7	
Waist circumference (cm)	74.7 ± 11.7	77.7 ± 12.3	71.6 ± 10.2	<0.001 *
Abdominal obesity (%)	20.2	25.1	15.0	0.001 ^#^
Sleeping ≥ 8 h/day (%)	39.3	47.2	31.3	<0.001 ^#^
Smokers (%)	11.3	10.7	11.7	0.019 ^#^
Population with food allergies (%)				<0.001 ^#^
To eggs	0.8	1.0	0.7	
To fish	1.7	0.3	2.7	
To fruits	3.0	2.0	4.0	
To vegetables	0.2	0.3	0.0	
To nuts	2.3	2.7	2.0	
Others	0.2	0.3	0.0	
Population with intolerances (%)				<0.001 ^#^
To gluten	1.5	2.3	0.7	
To lactose	8.2	5.4	11.0	
To fructose	1.2	0.7	1.3	

* Results are shown as mean values ± standard deviation, and differences between boys vs. girls were calculated by Student *t*-test. ^#^ Results are shown as percentages, and differences between boys vs. girls were calculated by chi-square. Data were adjusted by sociodemographic data.

**Table 2:** Preference for taste in adolescents by gender.

	All (n = 600)	Boys (n = 300)	Girls (n = 300)	*p*-Value *
Sweet	7.7 ± 1.5	7.8 ± 1.5	7.5 ± 1.4	0.018
Salty	5.4 ± 0.8	5.4 ± 0.8	5.4 ± 0.8	0.918
Bitter	2.7 ± 0.6	2.8 ± 0.6	2.7 ± 0.6	0.005
Sour	3.1 ± 0.8	3.0 ± 0.8	3.1 ± 0.8	0.150
Umami	5.6 ± 1.2	5.7 ± 1.1	5.5 ± 1.2	0.066

* Mean values ± standard deviation, boys vs. girls by Student *t*-test. Values have a range from (1) dislike extremely to (9) like extremely.

**Table 3:** Association between gender and preference for taste in adolescents.

	Boys (n = 300)	Girls (n = 300)	*p*-Value
Sweet	1.00 (ref.)	0.68 (0.49–0.94)	0.018
Salty	1.00 (ref.)	1.16 (0.72–1.88)	0.542
Bitter	1.00 (ref.)	0.76 (0.59–0.98)	0.036
Sour	1.00 (ref.)	1.20 (0.85–1.68)	0.301
Umami	1.00 (ref.)	0.93 (0.63–1.36)	0.698

Values are odds ratio (95% CI). Data were adjusted by sociodemographic data.

**Table 4:** Preference for taste in adolescents by age.

	11–13 Year-Old	14–15 Year-Old	16–18 Year-Old	*p*-Value *
All	n = 224	n = 212	n = 164	
Sweet	7.5 ± 1.4	7.7 ± 1.5	8.0 ± 1.5	0.001
Salty	5.3 ± 0.8	5.3 ± 0.8	5.5 ± 0.7	0.018
Bitter	2.8 ± 0.6	2.7 ± 0.6	2.7 ± 0.6	0.124
Sour	3.0 ± 0.7	3.0 ± 0.8	3.3 ± 0.8	0.131
Umami	5.5 ± 1.2	5.5 ± 1.2	6.0 ± 1.0	<0.001
Boys	n = 105	n = 113	n = 82	
Sweet	7.6 ± 0.5	7.7 ± 1.5	8.2 ± 1.5	0.010
Salty	5.3 ± 0.9	5.3 ± 0.9	5.5 ± 0.7	0.159
Bitter	2.9 ± 0.6	2.8 ± 0.6	2.8 ± 0.7	0.200
Sour	3.0 ± 0.7	3.0 ± 0.8	3.2 ± 0.8	0.094
Umami	5.5 ± 0.2	5.7 ± 1.2	5.7 ± 1.0	0.025
Girls	n = 119	n = 99	n = 82	
Sweet	7.3 ± 1.4	7.6 ± 1.4	7.8 ± 1.6	0.050
Salty	5.2 ± 0.8	5.4 ± 0.7	5.5 ± 0.7	0.070
Bitter	2.7 ± 0.6	2.7 ± 0.6	2.7 ± 0.6	0.401
Sour	3.0 ± 0.8	3.1 ± 0.7	3.4 ± 0.7	0.001
Umami	5.4 ± 1.2	5.4 ± 1.2	6.0 ± 1.0	<0.001

* Mean values ± standard deviation, differences between ages were assessed by ANOVA. Values have a range from (1) dislike extremely to (9) like extremely.

**Table 5:** Association between age and preference for taste in adolescents.

	11–13 Year-Old	14–15 Year-Old	16–18 Year-Old	*p*-Value
All				
Sweet	1.00 (ref.)	1.18 (1.00–1.38)	1.24 (1.05–1.46)	0.010
Salty	1.00 (ref.)	1.09 (0.85–1.41)	1.22 (0.89–1.68)	0.210
Bitter	1.00 (ref.)	0.62 (0.44–0.87)	0.54 (0.37–0.78)	0.001
Sour	1.00 (ref.)	0.89 (0.67–1.17)	1.28 (0.95–1.74)	0.107
Umami	1.00 (ref.)	1.04 (0.87–1.24)	1.39 (1.12–1.71)	0.642
Boys				
Sweet	1.00 (ref.)	1.22 (1.10–1.47)	1.48 (1.18–1.84)	0.001
Salty	1.00 (ref.)	0.97 (0.71–1.31)	1.17 (0.78–1.76)	0.822
Bitter	1.00 (ref.)	0.73 (0.48–0.98)	0.54 (0.33–0.87)	0.011
Sour	1.00 (ref.)	0.75 (0.54–1.01)	0.98 (0.67–1.45)	0.100
Umami	1.00 (ref.)	1.18 (0.95–1.47)	1.33 (0.93–1.75)	0.131
Girls				
Sweet	1.00 (ref.)	1.10 (0.90–1.35)	1.16 (0.95–1.42)	0.059
Salty	1.00 (ref.)	1.28 (0.91–1.80)	1.30 (0.88–1.92)	0.150
Bitter	1.00 (ref.)	0.48 (0.30–0.76)	0.46 (0.28–0.75)	0.002
Sour	1.00 (ref.)	1.04 (0.72–1.47)	1.76 (0.98–2.62)	0.834
Umami	1.00 (ref.)	1.02 (1.74–1.15)	1.37 (1.06–1.75)	0.014

Values are odds ratio (95% CI). Data were adjusted by sociodemographic data.

**Table 6:** Association between gender and food neophobia in adolescents.

	All (n = 600) *	Boys (n = 300)	Girls (n = 300)	*p*-Value
1. I am constantly sampling new and different foods (R)	2.75 ± 2.45	1.00 (ref.)	1.21 (0.88–1.67)	0.251
2. I don’t trust new foods	1.45 ± 2.25	1.00 (ref.)	1.18 (0.83–0.67)	0.368
3. If I don’t know what is in a food, I won’t try it	1.57 ± 2.29	1.00 (ref.)	0.90 (0.64–0.27)	0.538
4. I like foods from different countries (R)	3.43 ± 2.31	1.00 (ref.)	0.66 (0.47–0.93)	0.018
5. Ethnic food looks too weird to eat	1.57 ± 2.30	1.00 (ref.)	1.52 (1.08–2.51)	0.018
6. At dinner parties, I will try a new food (R)	1.90 ± 1.10	1.00 (ref.)	0.81 (0.57–0.17)	0.266
7. I am afraid to eat things I have never had before	1.90 ± 2.43	1.00 (ref.)	1.18(0.85–0.65)	0.313
8. I am very particular about the foods I will eat	2.26 ± 2.45	1.00 (ref.)	1.46 (1.06–2.02)	0.022
9. I will eat almost anything (R)	2.20 ± 2.44	1.00 (ref.)	0.59 (0.43–0.83)	0.002
10. I like to try new foods out of home (R)	2.88 ± 2.45	1.00 (ref.)	0.76 (0.55–1.05)	0.099
FNS total score	2.45 ± 1.10	1.00 (ref.)	0.96 (0.70–1.32)	0.806

(R): items were reversed; FNS: Food Neophobia Scale. * Mean values ± standard deviation. Gender-related columns are expressed as odds ratio (95% CI), as indicated in each column. *p*-value refers to odds ratio analysis. Data were adjusted by sociodemographic data.

**Table 7:** Association between age and food neophobia in adolescents.

	11–13 Year-Old	14–15 Year-Old	16–18 Year-Old	*p*-Value
1. I am constantly sampling new and different foods (R)	1.00 (ref.)	1.04 (0.72–1.52)	1.33 (0.89–2.01)	0.166
2. I don’t trust new foods	1.00 (ref.)	0.77 (0.62–0.97)	0.58 (0.37–0.93)	0.022
3. If I don’t know what is in a food, I won’t try it	1.00 (ref.)	1.18 (0.79–1.76)	0.90 (0.58–1.40)	0.419
4. I like foods from different countries (R)	1.00 (ref.)	0.72 (0.48–1.07)	1.20 (0.77–1.88)	0.426
5. Ethnic food looks too weird to eat	1.00 (ref.)	1.39 (0.93–2.07)	0.83 (0.53–1.31)	0.104
6. At dinner parties, I will try a new food (R)	1.00 (ref.)	1.04 (1.01–1.07)	1.94 (1.16–3.24)	0.011
7. I am afraid to eat things I have never had before	1.00 (ref.)	0.70 (0.48–1.03)	0.75 (0.50–1.13)	0.172
8. I am very particular about the foods I will eat	1.00 (ref.)	0.96 (0.66–1.39)	0.84 (0.56–1.27)	0.413
9. I will eat almost anything (R)	1.00 (ref.)	0.91 (0.62–1.33)	1.25 (0.83–1.87)	0.285
10. I like to try new foods out of home (R)	1.00 (ref.)	0.83 (0.57–1.20)	1.31 (0.87–1.98)	0.201
FNS total score	1.00 (ref.)	0.86 (0.73–1.01)	1.05 (0.88–1.25)	0.065

(R): items were reversed; FNS: Food Neophobia Scale. Age-related columns are expressed as odds ratio (95% CI), as indicated in each column. *p*-value refers to odds ratio analysis. Data were adjusted by sociodemographic data.

**Table 8:** Association between preferences for food tastes and food neophobia in adolescents.

	Sweet	Salty	Bitter	Sour	Umami
1. I am constantly sampling new and different foods (R)	1.01 (0.73–1.39)	1.75 (1.08–2.84)	1.37 (0.82–2.30)	1.72 (1.22–2.44) *	2.33 (1.55–3.50) *
2. I don’t trust new foods	0.95 (0.67–1.35)	0.95 (0.57–1.61)	1.02 (0.59- 1.78)	0.52 (0.35–0.77) *	0.58 (0.37–0.92) *
3. If I don’t know what is in a food, I won’t try it	0.81 (0.57–1.15)	1.17 (0.69–1.99)	1.61 (0.96–2.71)	0.65 (0.44–0.94) *	0.62 (0.40–0.95) *
4. I like foods from different countries (R)	1.49 (1.05–2.10) *	1.20 (0.73–1.99)	1.57 (0.87–2.82)	1.67 (1.14–2.44) *	2.64 (1.63–4.26) *
5. Ethnic food looks too weird to eat	0.67 (0.48–0.95) *	0.83 (0.50–1.38)	0.64 (0.35–1.15)	0.60 (41–0.88) *	0.38 (0.24–0.61) *
6. At dinner parties, I will try a new food (R)	1.19 (0.83–1.71)	1.06 (0.62–1.81)	1.30 (0.71–2.38)	1.47 (0.99–2.19)	1.63 (1.02–2.60) *
7. I am afraid to eat things I have never had before	0.87 (0.63–1.22)	1.16 (0.70–1.91)	0.88 (0.52–1.49)	0.69 (0.48–0.99) *	0.77 (0.52–1.15)
8. I am very particular about the foods I will eat	0.94 (0.68–1.29)	0.88 (0.55–1.42)	0.72 (0.43–1.21)	0.77 (0.55–1.09)	0.37 (0.24–0.56) *
9. I will eat almost anything (R)	0.89 (0.65–1.24)	1.27 (0.78–2.07)	1.72 (1.03–2.85) *	1.63 (1.16–2.29) *	2.17 (1.47–3.19) *
10. I like to try new foods out of home (R)	1.17 (0.85–1.62)	1.03 (0.63–1.66)	1.40 (0.83–2.36)	1.42 (1.00–2.01) *	1.71 (1.14–2.55) *
Unwillingness to change	1.06 (0.77–1.46)	1.28(0.80–2.07)	2.02(1.20–3.42) *	1.52(1.08–2.14) *	1.68 (1.14–2.47) *

Values are odds ratio (95% CI). * Differences (*p* < 0.05) in odds ratio analysis. Data were adjusted by sociodemographic data.

**Table 9:** Association between gender and neophobia towards food groups among adolescents.

	Boys (n = 300)	Girls (n = 300)	*p*-Value
Grains and tubers	1.00 (ref.)	1.09 (0.97–1.22)	0.163
Vegetables	1.00 (ref.)	1.10 (0.00–0.21)	0.042
Fruits	1.00 (ref.)	1.05 (0.95–1.16)	0.340
Nuts	1.00 (ref.)	0.99 (0.84–1.17)	0.899
Pulses	1.00 (ref.)	0.98 (0.82–1.17)	0.783
Dairies	1.00 (ref.)	1.02 (0.91–0.14)	0.730
Meats and vegetable substitutes	1.00 (ref.)	0.63 (0.47–0.86)	0.004
Eggs	1.00 (ref.)	2.22 (0.83–5.92)	0.111

Values are odds ratio (95% CI). *p*-value refers to odds ratio analysis. Data were adjusted by sociodemographic data.

**Table 10:** Association between age and neophobia towards food groups among adolescents.

	11–13 Year-Old	14–15 Year-Old	16–18 Year-Old	*p*-Value
Grains and tubers	1.00 (ref.)	0.83 (0.72–0.95)	1.12 (1.02–1.32)	0.008
Vegetables	1.00 (ref.)	0.84 (0.75–0.94)	0.90 (0.74–1.07)	0.004
Fruits	1.00 (ref.)	1.16 (1.06–1.28)	1.34 (1.15–1.56)	<0.001
Nuts	1.00 (ref.)	0.31 (0.03–3.03)	0.60 (0.18–2.01)	0.410
Pulses	1.00 (ref.)	0.57 (0.23–0.97)	0.57 (0.37–0.87)	0.010
Dairies	1.00 (ref.)	0.56 (0.13–2.38)	1.49 (0.48–4.63)	0.434
Meats and vegetable substitutes	1.00 (ref.)	0.95 (0062–1.46)	0.24 (0.97–1.60)	0.087
Eggs	1.00 (ref.)	1.05 (0.42–3.65)	1.20 (0.65–3.55)	0.911

Values are odds ratio (95% CI). Data were adjusted by sociodemographic data.

## Data Availability

There are restrictions on the availability of data for this trial due to the signed consent agreements around data sharing, which only allow access to external researchers for studies following the project’s purposes. Requestors wishing to access the trial data used in this study can make a request by emailing pep.tur@uib.es.

## Supplementary Materials

The following supporting information can be downloaded at: https://www.mdpi.com/article/10.3390/foods12203717/s1.
