# Supplementary material for: Assessing Food Preferences and Neophobias among Spanish Adolescents from Castilla–La Mancha
Source: Foods. 2023 Oct 10;12(20):3717. doi: 10.3390/foods12203717 (PMC10606373; doi:10.3390/foods12203717)
Supplement: Supplementary file 1 [file foods-12-03717-s001.zip › foods-2628410-supplementary.pdf]

Encuesta sobre aversiones alimentarias

Proyecto educativo de la Tesis Doctoral “Aversión alimentaria y estado nutricional”.
Departamento de Biología fundamental y Ciencias de la Salud.
Universitat de les Illes Balears.

Parte 1: datos generales de salud y medidas antropométricas

A rellenar por el investigador:

1. Edad (años): ________________
2. Selección su sexo: ◯ Mujer ◯ Hombre ◯ Otro
3. Estatura aproximada (en cm): _________________
4. Peso aproximado (en Kg): ______________________
5. Circunferencia de cintura (en cm): ________________

A rellenar por el alumno:

1. País de nacimiento: ____________________
2. ¿Cómo considera que es su estado de salud?

   ◯ Muy bueno

   ◯ Bueno

   ◯ Regular

   ◯ Malo

   ◯ Muy malo

   ◯ No sabe/no contesta

3. ¿Considera que su peso es adecuado?

   ◯ Si, me siento bien con mi peso

   ◯ No, me siento por encima de mi peso ideal

   ◯ No, me siento excesivamente delgado/a

4. ¿Sufre alguna enfermedad crónica?

   ◯ Diabetes mellitus

   ◯ Hipertensión

   ◯ Hipercolesterolemia

   ◯ Hipotiroidismo

   Otros ________________________

5. ¿Cuántas horas duerme al día entre semana?

   ◯ Menos de 8 horas al día

   ◯ Más de 8 horas al día

Parte 2: Hábitos de vida y alimentarios

6. ¿Fuma?
   - ◯ Si
   - ◯ No
7. ¿Cuántas veces a la semana consume carne procesada, es decir, hamburguesa, kebab, salchichas,…?
   - ◯ Menos de 2 veces a la semana
   - ◯ 2 o más veces a la semana
8. ¿Cuántas raciones de verduras u hortalizas consume al día? (las guarniciones o acompañamientos cuentan como media ración)
   - ◯ Menos de 2 raciones al día
   - ◯ 2 o más raciones al día (al menos una de ellas en ensaladas o crudas)

Parte 3: aversiones y preferencias alimentarias

9. Indique por favor, siendo 1 nada y 9 muchísimo, ¿cuánto le gusta el sabor de estos alimentos?

☐ Chocolate negro	☐ Frutos rojos	☐ Jamón curado
☐ Café con leche	☐ Chocolate con leche	☐ Patatas fritas (snack)
☐ Caramelo	☐ Limón	☐ Champiñón
☐ Vainilla	☐ Aceite de oliva	☐ Café (solo)
☐ Canela	☐ Mantequilla	☐ Caldo de pollo
		☐ Salsa de soja

10. Ahora, le presentamos una pareja de alimentos. Si le dieran a elegir qué producto escogería en base a su sabor. Seleccione uno de los dos:
    - ◯ Batido de multifrutas
    - ◯ Batido de chocolate
11. Entre la siguiente pareja de alimentos, ¿cuál le provoca más aversión?
    - ◯ Un plato de menestra de verduras
    - ◯ Un plato de caldereta de cordero.
12. Tacha los alimentos que NO TE GUSTEN de las siguientes tablas:

**Nuevos alimentos**		**Comida familiar**	
☐ Pulpo	☐ Bimi	☐ Salchichas	☐ Huevo de gallina
☐ Buey	☐ Huevo de codorniz	☐ Bacon	☐ Judías verdes
☐ Boquerones	☐ Edamame	☐ Atún de lata	☐ Queso de sándwich
☐ Queso de cabra	☐ Azukis	☐ Queso de oveja	☐ Lentejas
☐ Paté	☐ Rúcula	☐ Coliflor	☐ Lechuga

☐ Remolacha	☐ Aguacate	☐ Alcachofas	☐ Nueces
☐ Espárragos	☐ Sushi	☐ Setas	☐ Paella
☐ Tofu	☐ Kebbab	☐ Jamón cocido	☐ Albóndigas
☐ Tahini	☐ Fideos orientales	☐ Aceitunas	☐ Fideua de verduras
☐ Quinoa		☐ Macarrones	

13. De los siguientes grupos alimentarios, ¿hay algún alimento que no hayas probado o no te gusten? Tacha los que no hayas probado y marca con una **X** los que no te gusten.

FRUTAS

- ☐ Fresas
- ☐ Arándanos
- ☐ Plátano
- ☐ Melocotón
- ☐ Granada
- ☐ Higos
- ☐ Piña
- ☐ Otras________________

Grasas

- ☐ Aguacate
- ☐ Anacardos
- ☐ Almendras
- ☐ Pistachos
- ☐ Semillas de lino, chía,…
- ☐ Nueces
- ☐ Cacahuetes
- ☐ Otras________________

Carbohidratos

- ☐ Pan integral
- ☐ Arroz integral
- ☐ Pasta integral
- ☐ Avena
- ☐ Boniato / yuca
- ☐ Patata
- ☐ Quinoa
- ☐ Otras________________

Proteínas

- ☐ Huevos
- ☐ Garbanzos / lentejas
- ☐ Alubia blanca y pinta
- ☐ Guisantes
- ☐ Tofu / Tempeth
- ☐ Pescado _____________
- ☐ Carne_______________
- ☐ Caza ________________

Verduras	Lácteos y sustitutos
☐ Lechuga / hoja verde	☐ Leche / bebida vegetal
☐ Espinacas / acelgas	☐ Queso de oveja
☐ Tomate /pepino	☐ Yogur entero
☐ Berenjena /calabacín	☐ Yogur de soja
☐ Brócoli /coliflor	☐ Requesón
☐ Alcachofas	☐ Kéfir
☐ Espárragos	☐ Queso de cabra
☐ Otras_______________	☐ Otras_______________

14. ¿Te gustan los purés?
   - ◯ Si
   - ◯ No
15. ¿Eres intolerante a algún alimento o nutriente? Marca la respuesta adecuada
   - ◯ No soy intolerante a ningún alimento o nutriente
   - ◯ Gluten
   - ◯ Lactosa
   - ◯ Fructosa

   Otros_____________________
16. ¿Eres alérgico a algún alimento?
   - ◯ No soy alérgico a ningún alimento o nutriente
   - ◯ Huevos
   - ◯ Marisco/pescado
   - ◯ Frutas
   - ◯ Verduras
   - ◯ Frutos secos

   Otros________________________

Parte 4: evaluación psicológica para el cambio

17. Marque verdadero (**V**) o falso (**F**) a las siguientes cuestiones:
   - ◯ Pruebo constantemente nuevos alimentos y/o formas de preparación
   - ◯ No me gusta probar nuevos alimentos
   - ◯ Si no conozco un alimento, no lo pruebo
   - ◯ Me gustan los alimentos de diferentes culturas alimentarias o de diferentes países.

◯ En las reuniones con amigos y/o familiares, yo pruebo los alimentos nuevos que se me presentan

◯ Me da miedo probar alimentos que no he comido antes

◯ Soy muy especial para las comidas

◯ Yo puedo comer cualquier cosa

◯ Me gusta probar comida de otros países en restaurantes étnicos.

18. Cada una de las siguientes frases describe como podría sentirse una persona cuando aborda un problema en su vida. Marca con una X la frase que mejor describa tu comportamiento:

◯ Que yo sepa, no tengo problemas para cambiar

◯ Creo que puedo estar preparado para mejorar de alguna manera

◯ Estoy haciendo algo con los problemas que me han estado preocupando

◯ Me preocupa que dé un paso atrás con un problema que ya he cambiado, por eso considero que necesito ayuda

Parte 5: evaluación de la actividad física fuera de horas lectivas

19. Marca con una cruz la casilla correspondiente a las actividades físicas que realiza fuera del instituto. Indica el grado de intensidad con la que la realizas, siendo:

L → ligera   M → Moderado   F → Intensa o fuerte

◯ Pasear

◯ Andar de casa al instituto y del instituto a casa

◯ Trotar ("Jogging")

◯ Correr de 8-11 Km/h

◯ Excursiones por el campo

◯ Bicicleta

◯ Bailar

◯ Aerobic / zumba

◯ Hacer ejercicio en el gimnasio

◯ Hacer ejercicio en casa

◯ Nadar

◯ Baloncesto

◯ Patinar

◯ Montar a caballo

◯ Tenis / Padel

◯ Fútbol

◯ Otros ___________________________________
